# Laser-Induced and MOF-Derived Metal Oxide/Carbon Composite for Synergistically Improved Ethanol Sensing at Room temperature

**DOI:** 10.1007/s40820-024-01332-5

**Published:** 2024-02-09

**Authors:** Hyeongtae Lim, Hyeokjin Kwon, Hongki Kang, Jae Eun Jang, Hyuk-Jun Kwon

**Affiliations:** 1 Department of Electrical Engineering and Computer Science, DGIST, Daegu, 42988 South Korea; 2 Convergence Research Advanced Centre for Olfaction, DGIST, Daegu, 42988 South Korea

**Keywords:** Metal–organic frameworks, Metal oxide, Carbon composite, Laser, Gas sensor

## Abstract

Advancements in sensor technology have significantly enhanced atmospheric monitoring. Notably, metal oxide and carbon (MO_*x*_/C) hybrids have gained attention for their exceptional sensitivity and room-temperature sensing performance. However, previous methods of synthesizing MO_*x*_/C composites suffer from problems, including inhomogeneity, aggregation, and challenges in micropatterning. Herein, we introduce a refined method that employs a metal–organic framework (MOF) as a precursor combined with direct laser writing. The inherent structure of MOFs ensures a uniform distribution of metal ions and organic linkers, yielding homogeneous MO_*x*_/C structures. The laser processing facilitates precise micropatterning (< 2 μm, comparable to typical photolithography) of the MO_*x*_/C crystals. The optimized MOF-derived MO_*x*_/C sensor rapidly detected ethanol gas even at room temperature (105 and 18 s for response and recovery, respectively), with a broad range of sensing performance from 170 to 3,400 ppm and a high response value of up to 3,500%. Additionally, this sensor exhibited enhanced stability and thermal resilience compared to previous MOF-based counterparts. This research opens up promising avenues for practical applications in MOF-derived sensing devices.

**Supplementary Information:**

The online version contains supplementary material available at 10.1007/s40820-024-01332-5.

## Highlights

- Metal oxide and carbon hybrids (MO_x_/C) were micropatterned very rapidly and energy efficiently by direct laser writing.
- Metal-organic framework was the ideal precursor for fabricating homogeneous MO_x_/C hybrids due to regularly spaced metal ions and organic ligands.
- The fabricated sensor not only demonstrated broad-range gas sensing capability for ethanol gas (170-3,400 ppm) but also exhibited exceptional sensitivity, rapid response and recovery, selectivity, linearity, and thermal stability.

**Supplementary Information:**

The online version contains supplementary material available at 10.1007/s40820-024-01332-5.

## Graphical abstract

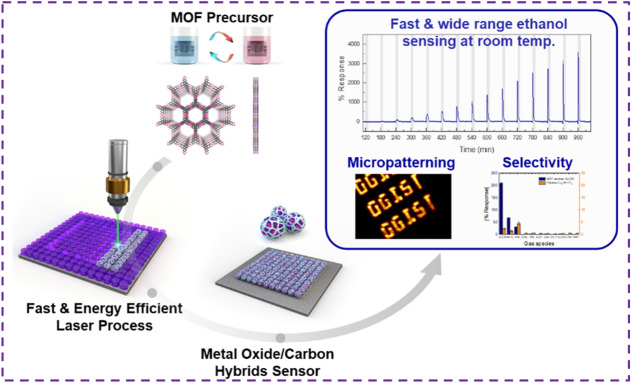

## Introduction

In recent years, gas sensor technology has played a pivotal role in characterizing our understanding of atmospheric conditions. Notably, ethanol is extensively utilized in various fields, including medical therapeutics, chemical engineering processes, and the food industry. However, it can easily volatilize into the ambient atmosphere, leading not only to an explosion risk but also to potential physiological responses, such as respiratory irritation, narcotic effects, and impaired perception [1]. Consequently, there is a pressing need to develop high-performance ethanol sensors characterized by sensitivity, reliability, low power consumption, cost-effectiveness, and rapid response/recovery, as well as homogeneous fabrication.

To meet these requirements, researchers have widely explored novel functional sensing materials, including MXene, transition metal dichalcogenides (TMDs), metal–organic frameworks (MOFs), and graphene-based nanomaterials, to increase porosity and surface area, design guest–host interactions, and create heterostructures [2, 3]. Among the various sensing materials, hybrid structures of metal oxide and carbon (MO_*x*_/C) are extensively attractive ethanol sensing materials due to their room-temperature sensing capabilities [4] and exceptional sensitivity due to carbon acting as a transducer platform that very sensitively reflects electrical charge interaction at the MO_*x*_ interface upon adsorption of analyte gas molecules [5–9]. In addition, the MO_*x*_/C structure exhibits remarkable stability and reliability, which are characteristics of metal oxide-based sensors, distinguishing it from other emerging materials. However, in terms of device fabrication, ex situ transferred MO_*x*_/C composites are limited by challenges, such as inhomogeneity, aggregation, and difficulties in patterning during the solution process (drop casting, screen printing, inkjet printing, etc.) [6].

Herein, we present a novel approach that utilizes a direct laser writing process on MOFs that are ideal precursors for MO_*x*_/C. MOFs inherently possess regularly spaced metal ions and organic linkers, making them suitable for generating homogeneous hybrid structures composed of carbonaceous and metallic components [10–12]. The flexibility in chemical composition, structural diversity, scalability, and extremely high surface area renders MOFs favorable as precursors for designing derivatives with customizable components and structures [13–15]. Several pioneering researchers have recently presented the concept of high-performance gas sensors based on MOF-derived metal oxides subjected to high-temperature pyrolysis [16, 17]. However, the thermal process is not energy efficient, has limitations in patterning due to isotropism, and requires a longer time in the microfabrication process. On the other hand, direct laser irradiation can enable rapid, energy-efficient programmable micropatterning [18]. The MOF-derived MO_*x*_/C film prepared through a laser process in this study displays a broad range of sensing capabilities for ethanol gas, coupled with rapid response and recovery times even at room temperature. Furthermore, superior stability and thermal resistance were observed compared to pristine MOF-based sensors. Consequently, our study provides key strategies to realize high-performance MO_*x*_/C gas sensors in practical applications.

## Experimental Section

### Materials

All chemicals were obtained from commercial sources and used without further purification. Copper(II) acetate (99.999%) and 2,3,6,7,10,11-hexahydroxytriphenylene (HHTP) ligand (95%) were purchased from Alfa Aesar and Acros Organics, respectively.

### Fabrication Process

#### Cu_***3***_HHTP_***2***_ MOF Formation by Layer-by-Layer (LbL) Process

Cu_3_HHTP_2_ MOF was grown on Si/SiO_2_ substrate using the LbL process. The Si/SiO_2_ substrate cleaned with Piranha solution was alternatively soaked in an ethanolic solution of 1 mM copper acetate and 0.1 mM 2,3,6,7,10,11-hexahydroxytriphenylene with retention times of 20 and 40 min, respectively. After each soaking cycle, the substrate was washed with ethanol to remove the residual reactants. The trigonal HHTP linker binds to the square planar Cu^2+^ ions to form an extended two-dimensional hexagonal layer in the *ab* plane. Through repeated LbL cycles, MOFs are stacked along the *c*-axis with a 1D open channel. The number of soaking cycles was 15, and the process was accurately automated by using a rotary dip coater (Nadetech ND-R Rotary Dip Coater). Then, Cu_3_HHTP_2_ MOF on Si/SiO_2_ substrate was rinsed with acetone and isopropyl alcohol and dried in a vacuum oven.

#### Direct Laser Irradiation on Cu_***3***_HHTP_***2***_

Cu_3_HHTP_2_ film on Si/SiO_2_ substrate was directly irradiated by Gaussian continuous-wave laser (green, 532 nm). The micropatterning process was implemented by *X*–*Y*–*Z* direction precision Aerotech stage and actuator. The irradiation power and scan speed were 0.4 W and 0.1 mm s^−1^, respectively. The power was controlled through a combination of a polarizing beam splitter and a waveplate. The distance between the objective lens and the Cu_3_HHTP_2_ film was accurately controlled by adjusting the Z-stage height to maintain the in-focus state.

### Characterization

#### Analytical Characterization

The morphologies of Cu_3_HHTP_2_ and MOF-derived CuO/C were observed by field emission scanning electron microscopy (FE-SEM, Hitachi SU8020). The ultrahigh-resolution transmission electron microscopy (TEM) was performed using a ThermoFisher Themis Z TEM instrument. For the preparation of TEM samples, the focused ion beam (FIB, Helios NanoLab G3 UC) system was used. Note that Cu_3_HHTP_2_ was passivated by aluminum and amorphous carbon for energy-dispersive X-ray spectroscopy (EDS) and imaging analysis, respectively. High-resolution Raman spectra and mapping images were obtained by employing a Renishaw inVia Qontor system using 532 nm laser excitation with a laser power of 5 mW. A Nicolet Continuum infrared microscope (Thermo Scientific) was used to collect the Fourier transform infrared (FT-IR) spectra. X-ray diffraction (XRD) patterns of Cu_3_HHTP_2_ MOFs were recorded on an Empyrean X-ray diffractometer (Malvern Panalytical) with Cu K_*α*_ radiation (λ = 1.54056 Å). X-ray photoelectron spectroscopy (XPS) was performed using an ESCALAB 250Xi system (Thermo Scientific). The time of flight secondary ion mass spectrometry (ToF–SIMS) was conducted by TOF–SIMS 5–100 (Ion-tof) instrument. A primary beam with bismuth (Bi) was applied for spectrometry (30 keV, 0.9 pA). These analyzes were performed at the DGIST Center for Core Research Facilities (CCRF).

#### Testing of Gas Sensing Performance

The chemiresistive response was measured on a custom-made gas sensing test system. Application of DC voltage (1 V) and measurement of current were implemented by a semiconductor analysis system (Keithley 4200, Keithely 3706A, and KEYSIGHT B2902A). The gas flow was controlled by a mass flow controller (M3030VA, Line Tech).

The response of the gas sensor was calculated using Eq. (1):

1 $${\text{Response}}=\left({R}_{0}-R\right)/{R}_{0}\times 100 \left(\%\right)$$

where *R* is the resistance after exposure of the target gas, *R*_0_ is the initial resistance of the sensor.

The response time was defined as the time for the resistance level to increase from the baseline signal to 90% of the maximum resistance change. Similarly, the recovery time was defined as the time required for the resistance level to decrease from the maximum resistance change to 10% of the maximum resistance change.

## Results and Discussion

### Fabrication of MOF-Derived Metal Oxide/Carbon Hybrids by Laser Process

**Fig. 1:**
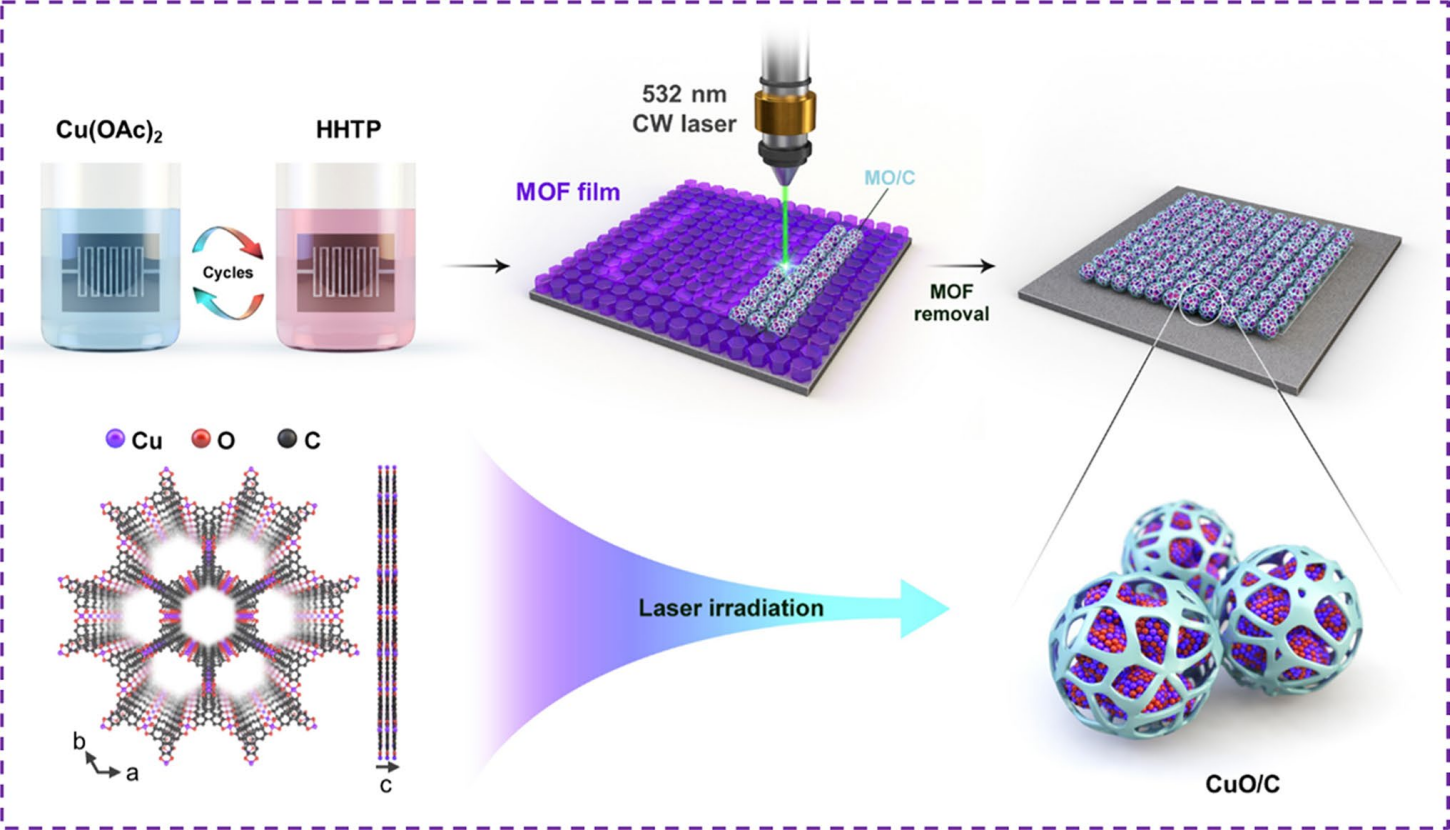
Schematic representation of the fabrication of the MOF-derived metal oxide/carbon composite (MO_*x*_/C) by a laser process

Figure 1 depicts the fabrication process and a structural schematic of the MOF-derived MO_*x*_/C composite sensor prepared by laser irradiation. Initially, a Cu_3_HHTP_2_ MOF was formed by a LbL process on a Si/SiO_2_ substrate with interdigitated electrodes (13 μm width and 7 μm gap). An optimized LbL process was conducted through successive immersion of a functionalized substrate in an ethanolic solution of metal cations and the HHTP ligand under ambient conditions. The trigonal HHTP ligand coordinates with square planar Cu^2+^ ions, forming an extended two-dimensional hexagonal layer in the *ab* plane [19]. This liquid-phase epitaxial process allows for precise control over the MOF film thickness compared to other coating techniques, such as slurry coating, solvothermal growth, or drop-casting [2, 20, 21]. The fabrication approach without the transfer process is also free from contamination issues, preventing the incorporation of impurity particles that can cause thermal damage during laser irradiation [22]. Moreover, Cu_3_HHTP_2_ is a well-established two-dimensional semiconducting MOF capable of gas sensing even in the pristine state [23]. Therefore, Cu_3_HHTP_2_ was selected as a proof of concept because it easily facilitated a straightforward comparison of gas sensing performance with that of the laser-irradiated MO_*x*_/C composite. After the formation of the MOF, the Cu_3_HHTP_2_ film was irradiated by a focused 532 nm continuous-wave laser to form a composite of metal oxide and carbon, which was controlled in the *X*–*Y*–*Z* directions using a precision stage. This laser writing process enabled the programmable mask-free patterning of CuO/C with a minimum line width of 2 μm, which was difficult to achieve with the previous solution-based process. Finally, the nonirradiated MOF region was removed by immersion in a basic solution. Additionally, the produced copper oxide is a p-type oxide semiconductor. Compared to n-type oxide semiconductors (such as ZnO, SnO_2_, and TiO_2_), which were typically used in previous oxide gas sensor studies, it exhibits unique catalytic activity and offers advantages in selectively detecting gaseous molecules through high redox activity. For this reason, contributing to the study of relatively underresearched p-type oxides (which account for only 12.06% of all gas sensor papers) will benefit the future expansion of the gas sensing library [24, 25].

### Analytical Characterization of MOF-Derived CuO/C

**Fig. 2:**
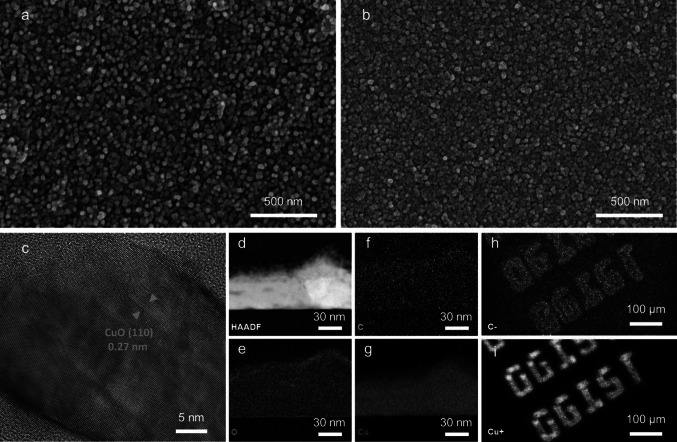
Characterization of the MOF-derived metal oxide/carbon composite. **a** SEM image of the pristine Cu_3_HHTP_2_ MOF. **b** SEM image of MOF-derived CuO/C after laser irradiation. **c** TEM image of MOF-derived CuO/C showing lattice fringes with an interplanar distance of 0.27 nm in the (110) crystal plane of CuO. **d**–**g** EDS-TEM images of MOF-derived CuO/C showing a uniform distribution of all elements; electron, carbon, oxygen, and copper maps, respectively. **h, i** ToF–SIMS mapping displaying the micropatterning of CuO/C by laser writing; carbon and copper

To demonstrate the successful growth of the MOF and the formation of CuO/C through the laser process, we implemented imaging analysis and analytical characterization. Figure 2a displays the SEM images of the Cu_3_HHTP_2_ MOF nanocrystals. The FT-IR spectroscopy spectrum and XRD pattern show successful growth of Cu_3_HHTP_2_, in good agreement with previous studies (Fig. S1) [26, 27]. After the laser calcination process, CuO/C nanoparticles were patterned on the substrates without delamination, perforation, or crack issues, as shown in Fig. 2b. Furthermore, the laser-induced accelerated heating and cooling of the MOF can prevent the formation of large and irregular particles during film formation. Thus, it is possible to retain porosity, resulting in enhanced sensing performance, including high sensitivity and fast response. The TEM image of CuO/C reveals distinct lattice fringes with interplanar distances of 0.27 nm in the (110) crystal plane of CuO, as shown in Fig. 2c [28]. The cross-sectional EDS results for CuO/C show uniform distributions of C, O, and Cu, confirming the formation of metal oxide and carbon compounds (Fig. 2d–g). Additionally, ToF–SIMS was introduced to show the programmable and selective laser patterning process (Fig. 2h, i). It shows the mapping of Cu and C along the patterned symbol of our institute.

**Fig. 3:**
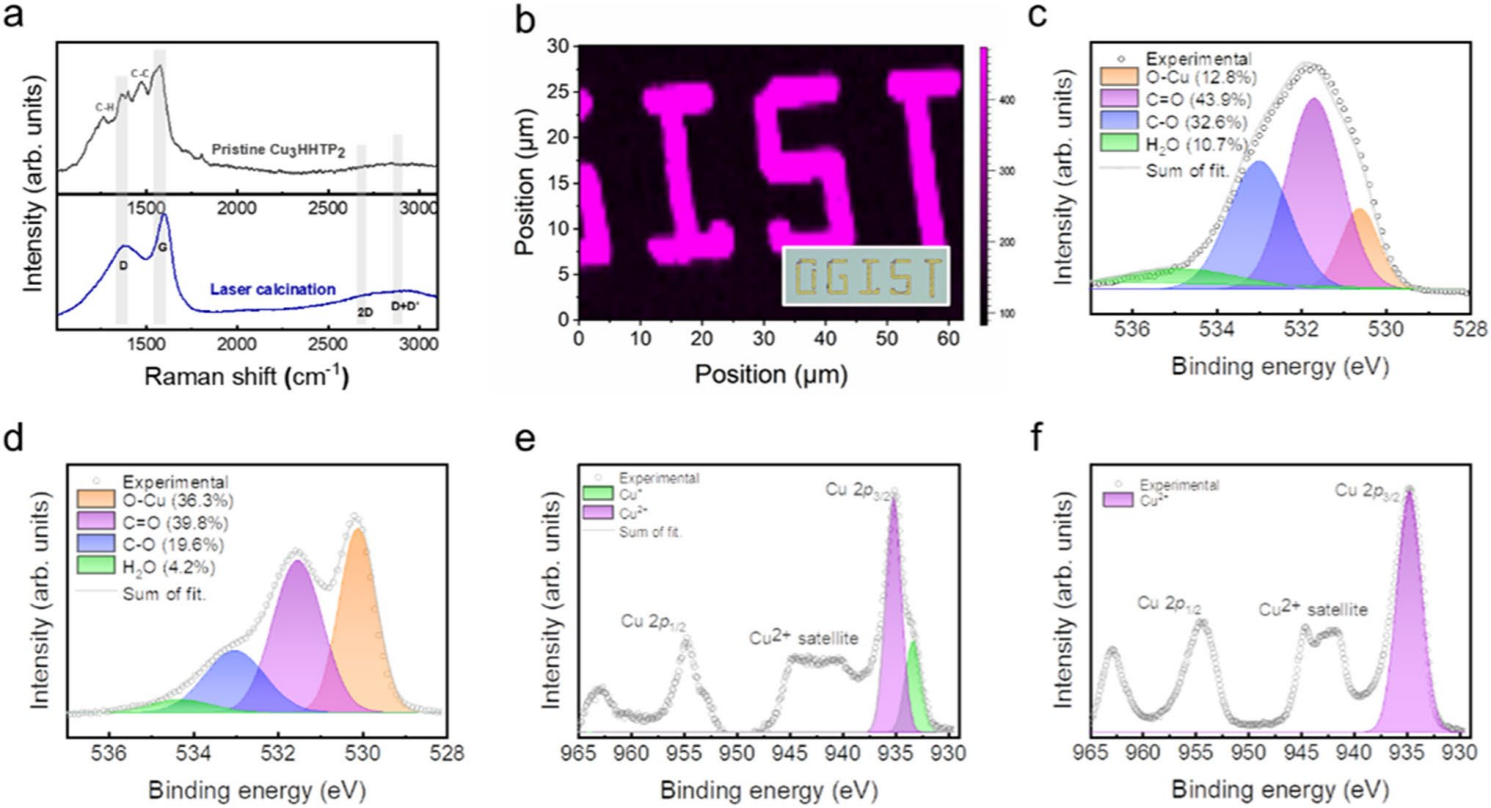
Spectroscopic analysis of the MOF-derived CuO/C composite. **a** High-resolution Raman spectra of pristine Cu_3_HHTP_2_ and the MOF-derived CuO/C composite. **b** Raman mapping of graphitic G bands in a laser-irradiated region. **c, d** XPS O 1*s* spectra of Cu_3_HHTP_2_ and the MOF-derived CuO/C composite. **e, f** XPS Cu 2*p*_3/2_ spectra of Cu_3_HHTP_2_ and the MOF-derived CuO/C composite

To further investigate the nature and chemical environment of MOF-derived CuO/C, spectroscopic analyses were performed. Figure 3a shows the high-resolution Raman spectra of pristine Cu_3_HHTP_2_ (upper) and CuO/C (bottom). In pristine Cu_3_HHTP_2_, the C-H in-plane bending modes (1,270 and 1,179 cm^−1^) and the stretching of aromatic C–C bonds (1,400, 1,468, and 1,547 cm^−1^) were observed, in addition to the G and D bands resulting from the graphitic platform of the aromatic triphenylene ligand [27]. After laser irradiation, the vibration peaks attributed to organic ligands disappeared, and only the D and G bands arising from graphitic carbon were observed. Additionally, the 2D band was preserved, which is the fingerprint signal of graphene and the second-order overtone of the D band [29]. However, the 2D band exhibited a lower intensity than the broad D + D’ peak, indicating that the resulting carbonaceous component is a multilayered and amorphous *sp*^2^ carbon structure. Similarly, several recent studies involving the annealing of MOFs have reported the coating of graphitic carbon on the surface of the resulting products [11, 12, 30]. During laser irradiation, localized heating caused the pyrolysis of organic ligands, resulting in the formation of Cu^2+^ ions. These ions then reacted with oxygen species in the atmosphere to form copper oxide [31]. Then, upon instant cooling, the thermally decomposed carbonaceous materials from the organic ligands (such as C_2_H_2_, CH_4_, and CO) formed a graphitic carbon layer on the surface of the oxide [30]. Additionally, high-resolution Raman mapping was performed to show the miniaturization of the laser patterning process. Figure 3b displays the spatial mapping of the peaks to baselines of graphitic G bands of CuO/C patterned with a 2 µm minimum line width. It should be noted that the patternable size of CuO/C in the laser writing process has the potential to be further reduced. Such miniaturization can be realized by optimizing parameters affecting the laser beam size, such as laser fluence, beam shaping, and the choice of objective lens.

The chemical environment and valence states of MOF-derived CuO/C were characterized by XPS. In the O 1*s* spectra, pristine Cu_3_HHTP_2_ can be deconvoluted into the main C=O peak (531.6 eV), the C-O peak (532.9 eV), and the O-Cu peak (530.5 eV), as shown in Fig. 3c. After laser irradiation, the ratio of C=O and C-O decreased due to the calcination of the organic ligand, resulting in the clear distinction of the O-Cu peak (Fig. 3d). In Fig. 3e, the Cu 2*p*_3/2_ peak of Cu_3_HHTP_2_ exhibited an asymmetric shape because the redox-active HHTP ligand capable of having multiple oxidation states exists in the semiquinonate and catecholate states, resulting in Cu^2+^ and Cu^+^ mixed-valency metal states [20, 27]. In contrast, laser-processed CuO/C clearly showed a Cu(II) valence state, which was characterized by a binding energy at 933.5 eV and a strong Cu^2+^ satellite, reflecting the influence of the intensive laser fluence during the patterning process (Fig. 3f) [32, 33].

### High-Performance Ethanol Monitoring

**Fig. 4:**
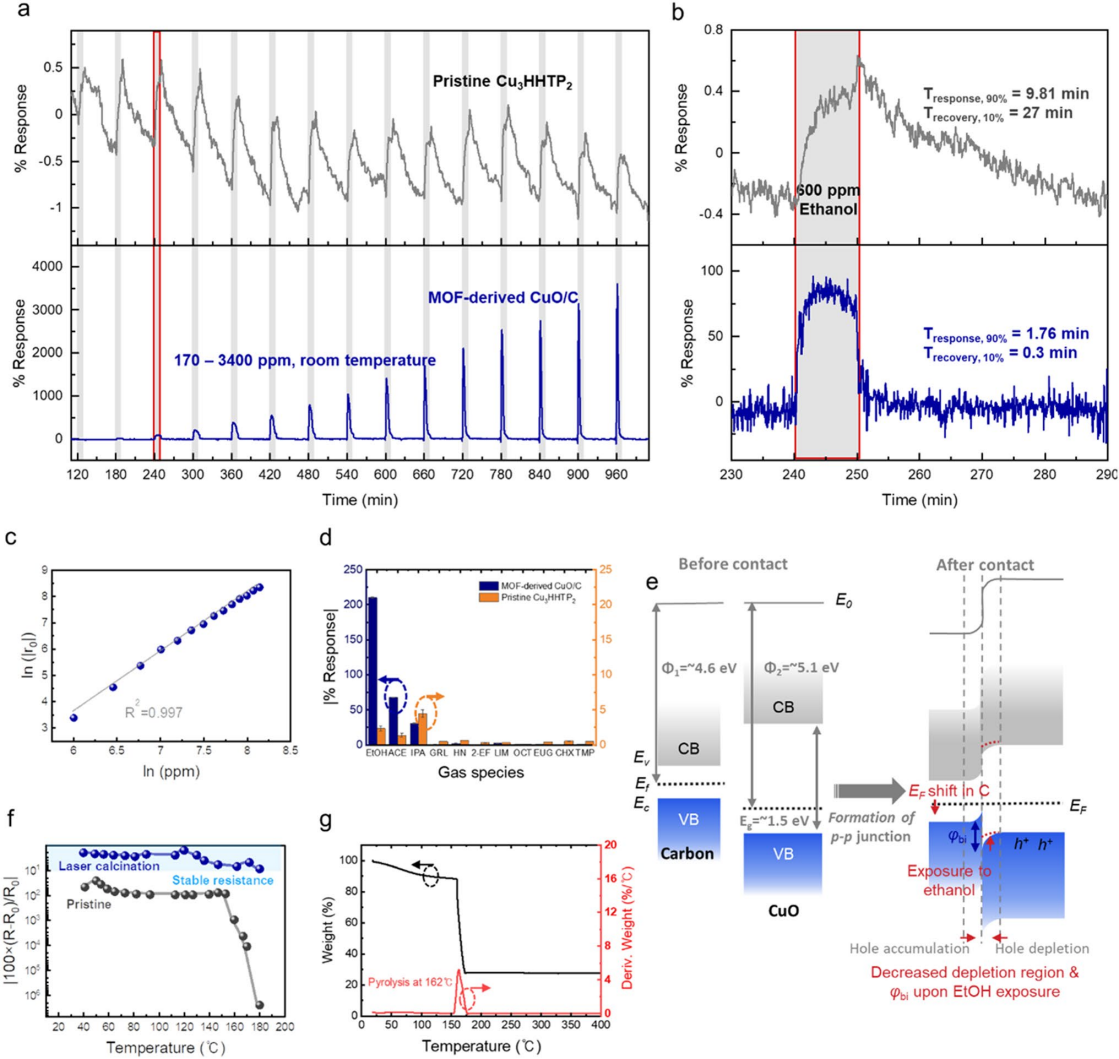
High-performance ethanol gas sensing. **a** Response and recovery curve with different ethanol concentrations (170–3,400 ppm) at room temperature. **b** Comparison of the response and recovery time of pristine Cu_3_HHTP_2_ and MOF-derived CuO/C under exposure to 600 ppm ethanol. **c** Linear correlation between the ln(response) and ln(concentration) of ethanol. **d** Response of CuO/C and Cu_3_HHTP_2_ to various analytes (volatile organic compounds and odorant molecules). **e** Energy band diagram illustrating the formation of a junction between carbon and copper oxide. **f** Change in electrical resistance after heating of pristine Cu_3_HHTP_2_ and MOF-derived CuO/C sensors. **g** Thermogravimetric analysis data of Cu_3_HHTP_2_

The sensing performance of MOF-derived CuO/C was assessed for ethanol gas detection at room temperature. Due to the dual threats of explosion hazards and physiological implications associated with ethanol, there is a critical need for rapid monitoring of ethanol concentrations over a broad range [1]. In Fig. 4a, the ethanol sensing performance of pristine Cu_3_HHTP_2_ and laser-calcined CuO/C sensors was compared. Upon exposure to 170 to 3,400 ppm (10 min exposure—50 min recovery cycle), the CuO/C sensor could rapidly detect ethanol gas over a wide range that spans the permissible exposure limits for the general industry set by the Occupational Safety and Health Administration (1,000 ppm) and 10% of the lower explosion limit (3,300 ppm) for explosion alarm systems. In particular, CuO/C displayed a much faster response and recovery time (105 and 18 s, respectively) than the pristine Cu_3_HHTP_2_ MOF (Fig. 4b). In addition to exhibiting fast recovery and a stable baseline signal, it was free from the baseline-shift issue common in previous chemiresistor-type sensors [34, 35]. These superior properties ensure reliable operation that can distinguish gas concentrations even during prolonged device operation. The response at adsorption–desorption equilibrium was plotted against the concentration. It revealed a linear relationship between ln(response) and ln(concentration) (Fig. 4c), a typical feature of the Langmuir–Freundlich chemical adsorption model [36]. This linear relationship allows for the accurate and straightforward readout of gas concentrations. The theoretical limit of detection (LoD) was also calculated using two methods. The first was obtained by setting the concentration value corresponding to a 10% response in a linear approximation, which is 5.464 ppm [19]. The second method, based on IUPAC recommendations, calculates it as 3 × (root mean square of noise/slope of regression), resulting in an LoD of 1.109 ppm [27].

We further investigated selectivity, which is one of the crucial requirements for gas sensor operation in the real world. As shown in Fig. 4d, the response value under 900 ppm ethanol exposure was 213%, showing the greatest response compared to the same concentration of other VOCs and odorant molecules in the air (acetone, isopropanol, geraniol, 1-heptanol, 2-ethylfenchol, D-limonene, octanal, eugenol, cis-3-hexenol, and 2,3,5-trimethylpyrazine). The excellent response performance of CuO/C could be elucidated through the energy band diagram in Fig. 4e. When copper(II) oxide, which has a bandgap energy of 1.2–1.5 eV and a work function of 5.0–5.1 eV, forms a junction with carbon, a built-in potential results [4, 37, 38]. Although pinpointing the energy structure of the carbon product from the laser calcination process can be challenging, the product is inferred to have a small bandgap and a work function of 4.6 eV. This is based on energy structure studies of laser-induced graphene and reduced graphene oxides, which exhibit similar few-layered *sp*^2^ carbon structures (Fig. S2) [27, 39, 40]. Hence, numerous p-p junctions are formed in CuO/C nanoparticles. During gas adsorption, as the Fermi level of carbon decreases, this leads to a reduction in the energy barrier. Consequently, there is an increase in the conduction of holes, which are the primary carriers of copper oxides. This results in a sensitive conversion into electrical signals.

The strong interaction between CuO/C and ethanol can be attributed to the influence of the surface graphitic carbon layer formed from the localized rapid heating and cooling in the laser process. The graphitic carbon, which can be observed in Raman spectra (Fig. 3a) and reported in recent studies on laser-irradiated metal–organic frameworks [30, 31], offers a high density of defect sites and functional groups. To investigate the chemical environment of the formed carbon, we analyzed the XPS C 1*s* spectrum of MOF-derived CuO/C, as shown in Fig. S3. Interestingly, besides the C–C peak, which is the basal plane of carbon in the CuO/C composite, strong intensities of C–O and C=O peaks were observed. These peaks imply the presence of a large number of functional groups on the carbon surface that can form hydrogen bonds with the –OH of ethanol, such as hydroxyl (–OH) and carboxyl groups (–COOH). Furthermore, the reason for the high sensor selectivity toward ethanol, even among other alcohols, is that the α-C–H bond in ethanol is weaker than the α-C–H bond in secondary alcohols like isopropyl alcohol (IPA), leading to stronger interactions (hydrogen bonding) with the functional groups on the carbon surface [41]. Additionally, non-alcoholic VOCs like acetone and complex aromatic molecules have relatively high activation energies. Thus, the CuO/C sensor can enhance selectivity toward ethanol at room temperature.

The thermal stability of gas sensing materials not only enables their use in a wide range of applications, such as in factories or the automotive industry, but also ensures compatibility when integrated with other sensors or during subsequent fabrication stages [42]. Figure 4f displays the resistance changes after thermal heating for both pristine MOF and MOF-derived CuO/C. With calcination of the MOF ligand by a laser process, CuO/C showed little change in electrical resistance even when the temperature changed from room temperature to 200 °C. On the other hand, in the case of pristine Cu_3_HHTP_2_, ligand pyrolysis occurred at 160 °C, as indicated in the thermogravimetric analysis (TGA) plot (Fig. 4g), thereby restricting its application in environments with demanding temperature endurance requirements. Furthermore, as shown in Fig. S4, we demonstrated the long-term stability and reusability by examining the changes in the ethanol sensing performance four weeks after the initial experiment.

## Conclusions

In conclusion, we have successfully engineered a MOF-derived CuO/C sensor that can swiftly detect a broad spectrum of ethanol levels using a laser irradiation process. This sensor demonstrates a prompt response and recovery under exposure to ethanol gas at room temperature, coupled with a consistent baseline current. We attribute this exceptional performance to the high surface area derived from the MOF structure. Moreover, the thermal stability of the sensor is enhanced due to the calcination of the ligand, which contrasts with the attributes of pristine MOF. From a device manufacturing standpoint, the micropatterning technique employing direct laser irradiation of MOFs presents several advantages. Conventional methods for producing MO_*x*_/C through solution-based processes or transfer methods have encountered challenges such as particle aggregation, patterning resolution restrictions, and material inhomogeneity. However, utilizing MOFs as a precursor and generating MO_*x*_/C directly via laser photothermal effects allows for uniform and intricate micropatterning. These insights pave the way for potential real-world applications in the domain of MOF-derived sensing devices.

## Supplementary Information

Below is the link to the electronic supplementary material.

Supplementary file1 (PDF 269 KB)
