# Supplementary material for: Laser-Induced and MOF-Derived Metal Oxide/Carbon Composite for Synergistically Improved Ethanol Sensing at Room temperature
Source: Nanomicro Lett. 2024 Feb 9;16:113. doi: 10.1007/s40820-024-01332-5 (PMC10858016; doi:10.1007/s40820-024-01332-5)
Supplement: Supplementary file 1 — Supplementary file1 (PDF 269 KB) [file 40820_2024_1332_MOESM1_ESM.pdf]

Supporting Information for

Laser-Induced and MOF-Derived Metal Oxide/Carbon Composite for Synergistically Improved Ethanol Sensing at Room temperature

Hyeongtae Lim[1,2], Hyeokjin Kwon[1,2], Hongki Kang[1], Jae Eun Jang[1], and Hyuk-Jun Kwon[1,2,*]

[1] Department of Electrical Engineering and Computer Science, DGIST, Daegu 42988, S. Korea

[2] Convergence Research Advanced Centre for Olfaction, DGIST, Daegu 42988, S. Korea

*Corresponding author. E-mail: hj.kwon@dgist.ac.kr (Hyuk-Jun Kwon)

Supplementary Figures

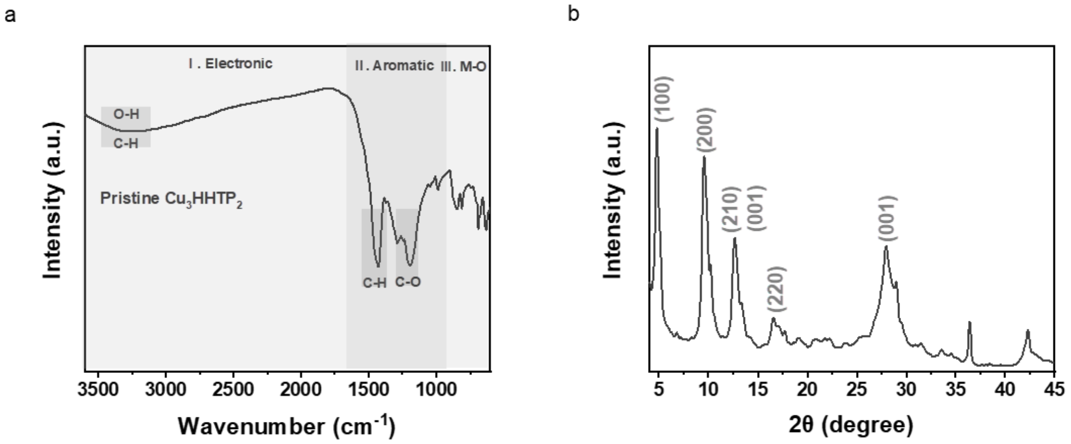

Fig. S1 **a** Fourier transform infrared (FT-IR) spectroscopy and **b** X-ray diffraction (XRD) pattern of Cu_3HHTP_2 MOF. The peaks of Cu_3HHTP_2 were in good agreement with previous studies [S1, S2]

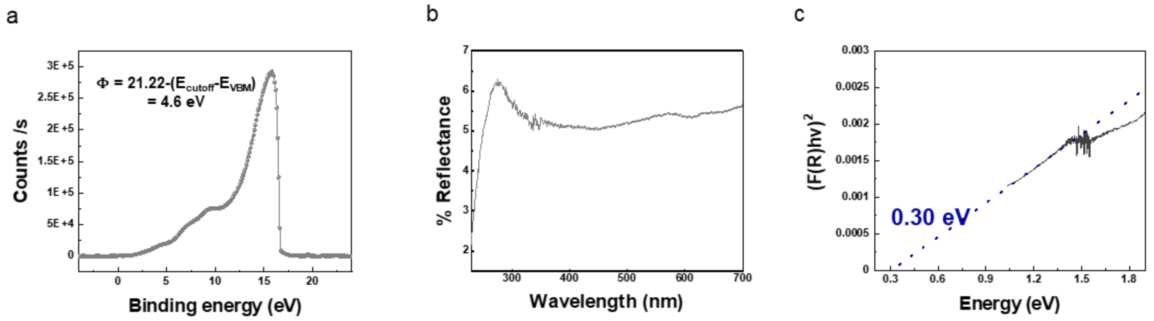

Fig. S2 Estimation of the energy band diagram of laser-induced graphene. **a** UPS spectrum and work function calculation. **b** UV-vis reflectance spectrum and **c** Tauc plot

Direct investigation of the band structure of the carbon element in the CuO/C composite was challenging. Instead, we hypothesized that this carbon element would have a similar chemical environment to the few-layered sp^2 carbon content reduced graphene oxides (rGO) as observed in Raman spectroscopy and XPS studies. Therefore, we conducted the extraction process of the energy band diagram of rGO-like laser-induced graphene obtained from direct laser irradiation on the carbon-containing substrate (PI) in a manner similar to our previous research [S2].

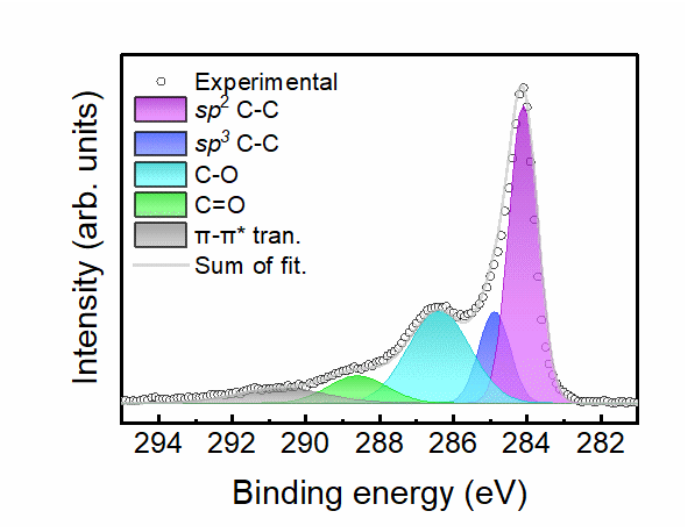

Fig. S3 XPS C 1*s* spectrum of MOF-derived CuO/C

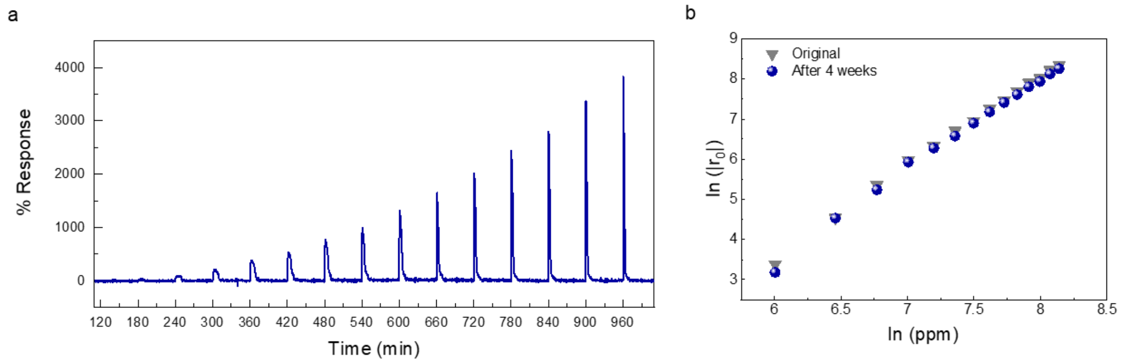

Fig. S4 Ethanol monitoring performance after four weeks. The sensor for the stability test was stored at room temperature and in air condition. **a** Dynamic response curve with different ethanol concentrations (170-3400 ppm) at room temperature. **b** Linear correlation between the ln(response) and ln(concentration)

Supplementary References

[S1] K. W. Nam, S. S. Park, R. dos Reis, V. P. Dravid, H. Kim et al., Conductive 2d metal-organic framework for high-performance cathodes in aqueous rechargeable zinc batteries. Nat. Commun. **10**(1), 4948 (2019). https://doi.org/10.1038/s41467-019-12857-4

[S2] H. Lim, H. Kwon, H. Kang, J. E. Jang, H.-J. Kwon. Semiconducting MOFs on ultraviolet laser-induced graphene with a hierarchical pore architecture for NO_2 monitoring. Nat. Commun. **14**(1), 3114 (2023). https://doi.org/10.1038/s41467-023-38918-3
